# The hierarchical stability of the seven known large size ratio triple asteroids using the empirical stability parameters

**DOI:** 10.1007/s10509-013-1663-3

**Published:** 2013-10-26

**Authors:** Xiaodong Liu, Hexi Baoyin, Franck Marchis

**Affiliations:** 1 School of Aerospace, Tsinghua University, Beijing, 100084 China; 2 Carl Sagan Center, SETI Institute, 189 Bernardo Avenue, Mountain View, CA 94043 USA

**Keywords:** Satellites of asteroids, Celestial mechanics, Asteroids, dynamics

## Abstract

In this study, the hierarchical stability of the seven known large size ratio triple asteroids is investigated. The effect of the solar gravity and primary’s *J*_2_ are considered. The force function is expanded in terms of mass ratios based on the Hill’s approximation and the large size ratio property. The empirical stability parameters are used to examine the hierarchical stability of the triple asteroids. It is found that the all the known large size ratio triple asteroid systems are hierarchically stable. This study provides useful information for future evolutions of the triple asteroids.

## Introduction

The triple asteroids are newly found systems in the Solar System. The system (87) Sylvia is the first known triple asteroid system to be confirmed in 2004, for which the outer satellite Romulus was discovered in 2001 using Keck (Brown and Margot 2001; Margot and Brown 2001) and was also detected in HST images (Storrs et al. 2001), and the inner satellite Remus was discovered in 2004 using adaptive optics system on the Very Large Telescope (Marchis et al. 2005). Recently, Fang et al. (2012) derived the masses, orbits and primary’s oblateness of (87) Sylvia system, and examined the short-term and long-term stability of the orbits of two satellites numerically. At the time of writing this paper, nine triple asteroids (including two Kuiper Belt objects) have been identified, namely, (45) Eugenia (Marchis et al. 2010; Beauvalet et al. 2011), (87) Sylvia (Marchis et al. 2005; Fang et al. 2012), (93) Minerva (Marchis et al. 2011, 2013), (216) Kleopatra (Descamps et al. 2011), (136617) 1994 CC (Fang et al. 2011; Brozović et al. 2011), (153591) 2001 SN263 (Fang et al. 2011), (136108) Haumea (Ragozzine and Brown 2009), (47171) 1999 TC_36_ (Benecchi et al. 2010), and (3749) Balam (Marchis et al. 2008, 2012). The first seven are large size ratio triple asteroids, while the last two triple asteroids have their central components of a comparable size and orbiting very close to each other.

In connection with these recently discovered triple asteroids, the hierarchical stability of the large size ratio triple asteroid system including the effect of the solar gravity and primary’s *J*_2_ is analyzed in this study. The system is said to be hierarchically stable if its hierarchical configuration is not altered over a time scale that is much longer than the basic periods of the system (Milani and Nobili 1983; Walker and Roy 1983). Thus, either orbit crossing or escape of the bodies from the system is not possible. Although the four-body that consists of the triple asteroids and the Sun is non-integrable, the hierarchical stability can be examined using the Walker-Roy empirical stability criteria (Walker 1983a; Walker and Roy 1983). In this study, only the seven large size ratio triple asteroids are concerned because it is convenient to expand the force function in terms of mass ratios. The effect of the primary’s *J*_2_ is added to the traditional empirical stability parameters. In previous studies, numerical simulations were performed to investigate orbital stability of (87) Sylvia (Winter et al. 2009; Frouard and Compère 2012; Fang et al. 2012) and stability regions around (153591) 2001 SN263 (Araujo et al. 2012).

For clarity, this paper is structured as follows: in Sect. 2, the model of the large size ratio triple asteroid system involving the Sun in the four-body problem is described, and the force function is expanded in terms of mass ratios. In Sect. 3, the empirical stability parameters are used to examine the hierarchical stability of the triple asteroids. Finally, our conclusions are presented in Sect. 4.

## Model of the large size ratio triple asteroid system involving the solar gravity and primary’s *J*_2_ in the four-body problem

The large size ratio triple asteroids involving the Sun in the four-body problem consists of a nonspherical primary with mass *m*_1_, two small satellites (also known as moonlets) with masses *m*_2_ and *m*_3_, and the Sun with mass *m*_4_. The simple four-body hierarchy instead of the double hierarchy is adopted here (Milani and Nobili 1983; Walker 1983a; Walker and Roy 1983). In other words, these four bodies are arranged that the body *m*_*i*_ (*i*=2,3,4) is in orbit about the barycenter of all of its inner bodies, i.e., *m*_1_,…,*m*_*i*−1_.

Hill’s approximation is used here since the triple asteroids are close to each other, their center of mass is far away from the Sun, and the mass of the Sun dominates the four-body system (Hamilton and Krivov 1997; Scheeres 1998). Additionally, for the large size ratio triple asteroids, the mass of the primary is much larger than its moonlets, i.e. *m*_1_≫*m*_2_,*m*_3_. Based on the previous study (cf. Walker 1983a, the equations before Eq. (17)) and adding the effect of *J*_2_, the equations of motion in the expansion of the mass ratios are written as

1 $$\begin{aligned} \ddot{\boldsymbol{\rho}}_{i} =& GM_{i}\boldsymbol{\nabla}_{i} \Biggl\{ \frac{1}{\rho_{i}} \Biggl[ 1 - \frac{J_{2}R_{e}^{2}P_{2} ( \sin \varphi_{i} )}{\rho_{i}^{2}} \\ &{} + \sum_{k = 1}^{i - 1} \frac{m_{k}}{M_{i - 1}} \alpha_{ki}^{2}S_{ki}' + \sum _{l = i + 1}^{n} \frac{m_{l}}{M_{i - 1}}\alpha_{il}^{3}S_{il}' \Biggr] \Biggr\} , \end{aligned}$$

where ***ρ***_*i*_ is the vector from *M*_*i*−1_ to *m*_*i*_;*G* is the gravitational constant; $M_{i} = \sum_{j = 1}^{i} m_{j}$, located at the barycenter of the masses $m_{1}, \dots, m_{i}; \boldsymbol{\nabla}_{i} = \frac{\partial}{\partial \boldsymbol{\rho}_{i}}; \rho_{i} = \vert \boldsymbol{\rho}_{i} \vert ; \alpha_{ij} = \rho_{i}/\rho_{j}; S_{ij}' = \sum_{r = 0}^{\infty} \alpha_{ij}^{r}P_{r + 2} ( C_{ij} ); C_{ij} = \boldsymbol{\rho}_{i} {\cdot} \boldsymbol{\rho}_{j}/\rho_{i}\rho_{j}; P_{r}$ is the Legendre function of degree *r*;*J*_2_ is the primary’s oblateness coefficient; *R*_*e*_ is the primary’s reference radius; and *φ*_*i*_ is the latitude of *m*_*i*_ in the primary-fixed coordinate system. Taking the (216) Kleopatra system as an example, the primary is Kleopatra with the oblateness coefficient *J*_2_=0.6, and two moonlets are S/2008 ((216)) 1 and S/2008 ((216)) 2 (Descamps et al. 2011). Therefore, the four-body system consists of the primary Kleopatra, S/2008 ((216)) 1, S/2008 ((216)) 1, and the Sun. Because the semi-major axis of the moonlet is usually several times larger than the primary’s reference radius (Richardson and Walsh 2006; Johnston 2013), the effects of higher harmonic expansions are negligible. Therefore, only *J*_2_ term is considered in this paper.

Note that Eq. (1) is different from Eq. (17) in Walker (1983a). The reason is that *m*_1_ dominates the whole system in Walker (1983a) while *m*_4_ dominates our system and *m*_1_ only dominates the large size ratio triple asteroids in the present paper.

Take *m*_1_ as the unit of the mass, the scaled masses *m*_2_, *m*_3_, and *m*_4_ are denoted as *μ*_2_, *μ*_3_, and *μ*_4_, respectively. Reminding of the large size ratio property (*m*_1_≫*m*_2_,*m*_3_) and the Hill’s approximation (*ρ*_4_≫*ρ*_2_,*ρ*_3_ and *m*_4_≫*m*_1_,*m*_2_,*m*_3_), the equations of motion take a compact form

2 $$\begin{aligned} &{\ddot{\boldsymbol{\rho}}_{2} = GM_{2}\boldsymbol{\nabla}_{2} \biggl[ \frac{1}{\rho_{2}} \biggl( 1 - \frac{J_{2}R_{e}^{2}P_{2} ( \sin \varphi_{2} )}{\rho_{2}^{2}} } \\ &{\phantom{\ddot{\rho}_{2} =}{}+ \mu_{3} \alpha_{23}^{3}S_{23}' + \mu_{4}\alpha_{24}^{3} \biggr) \biggr],} \end{aligned}$$

3 $$\begin{aligned} &{\ddot{\boldsymbol{\rho}}_{3} = GM_{3}\boldsymbol{\nabla}_{3} \biggl[ \frac{1}{\rho_{3}} \biggl( 1 - \frac{J_{2}R_{e}^{2}P_{2} ( \sin \varphi_{3} )}{\rho_{3}^{2}} } \\ &{\phantom{\ddot{\rho}_{3} =}{} + \mu_{2} \alpha_{23}^{2}S_{23}'+ \mu_{4}\alpha_{34}^{3} \biggr) \biggr],} \end{aligned}$$

4 $$\begin{aligned} &{\ddot{\boldsymbol{\rho}}_{4} = GM_{4}\boldsymbol{\nabla}_{4} \biggl[ \frac{1}{\rho_{4}} \biggl( 1 - \frac{J_{2}R_{e}^{2}P_{2} ( \sin \varphi_{4} )}{\rho_{4}^{2}} } \\ &{\phantom{\ddot{\rho}_{4} =}{}+ \mu_{2} \alpha_{24}^{2} + \mu_{3}\alpha_{34}^{2} \biggr) \biggr].} \end{aligned}$$

Note that the expressions are expanded in terms of mass ratios due to the properties of the large size ratio triple asteroid systems, which is different from Walker and Roy (1983) where the expressions were expanded in terms of the ratios of the orbital radii.

## Hierarchical stability of the large size ratio triple asteroids

According to Walker and Roy (1983), in our problem at the collinear configuration *m*_1_–*m*_2_–*m*_3_–*m*_4_ where both the mutual perturbations of the bodies and the perturbation due to primary’s *J*_2_ are greatest. Note that this collinear configuration is only an extreme scenario in the mathematical sense. In this scenario, *C*_*ij*_=1 and *φ*_*i*_=0, so that *P*_*r*_(*C*_*ij*_)=1 and *P*_2_(sin*φ*_*i*_)=−1/2 ∀*r*,*i*,*j*; there is also the equation $S_{ij}' = 1/ ( 1 - \alpha_{ij} )$. Walker and Roy (1983) introduced the empirical stability parameters that are the sum of the disturbing terms at this configuration, when they expanded the force function in terms of the ratios of the orbital radii. In the four-body problem, the physical significance of each empirical stability parameter *Σ*_*i*_ (*i*=2,3,4) can be considered as the measure of the disturbance of the system’s other components on the orbit of *m*_*i*_ relative to *M*_*i*−1_; in order to ensure the hierarchical stability, the values of *Σ*_*i*_ must be less than unity and generally less than 10^−2^ (Walker and Roy 1983). Walker and Roy (1983) also pointed out that the eccentricity’s effect on the *Σ*_*i*_ parameters is small. Thus, the eccentricity’s effect is not considered in this study, just as Walker et al. (1980), Walker (1983b), and Veras and Armitage (2004) when they investigated the Hill stability of the triple systems using analytic methods. In our problem for the large size ratio triple asteroid systems, the empirical stability parameters *Σ*_2_, *Σ*_3_, and *Σ*_4_ at the collinear configuration *m*_1_–*m*_2_–*m*_3_–*m*_4_ can be expressed as

5 $$\begin{aligned} &{\varSigma_{2} = J_{2}R_{e}^{2}/2/ \rho_{2}^{2} + \mu_{3}\alpha_{23}^{3}/ ( 1 - \alpha_{23} ) + \mu_{4}\alpha_{24}^{3},} \end{aligned}$$

6 $$\begin{aligned} &{\varSigma_{3} = J_{2}R_{e}^{2}/2/ \rho_{3}^{2} + \mu_{2}\alpha_{23}^{2}/ ( 1 - \alpha_{23} ) + \mu_{4}\alpha_{34}^{3},} \end{aligned}$$

7 $$\begin{aligned} &{\varSigma_{4} = J_{2}R_{e}^{2}/2/ \rho_{4}^{2} + \mu_{2}\alpha_{24}^{2} + \mu_{3}\alpha_{34}^{2}.} \end{aligned}$$

### Without consideration of the solar gravity effect and primary’s *J*_2_

**Fig. 1:**
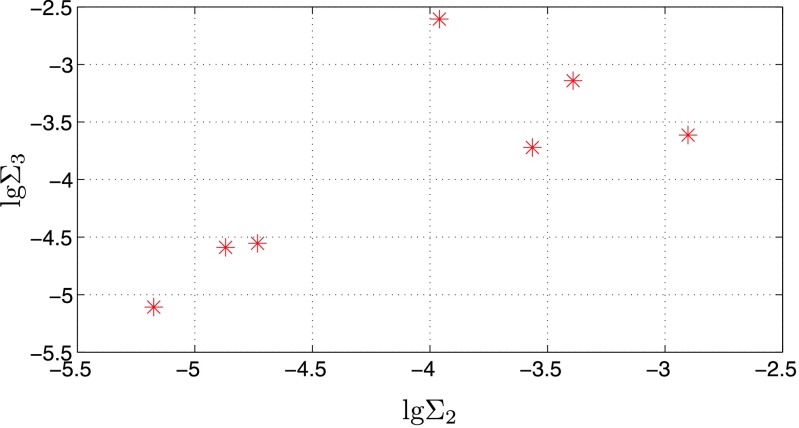
The values of empirical stability parameters *Σ*_2_ and *Σ*_3_ for the seven known large size ratio triple asteroids without considering the solar gravity effect and primary’s *J*_2_. The actual large size ratio triple asteroids cases are marked with asterisk

If the effect of the solar gravity and primary’s *J*_2_ is not taken into account, i.e. *μ*_4_=0, ***ρ***_4_=0, and *Σ*_4_=0, the system only consists of three masses. Table 1 lists ratios of masses and orbital radii for the seven known large size ratio triple asteroids. Based on Eqs. (5)–(6), the values of *Σ*_2_ and *Σ*_3_ for the seven known large size ratio triple asteroids are shown in Fig. 1 using the parameters listed in Table 1. It is easy to see that the values of *Σ*_2_ and *Σ*_3_ are all much less than 10^−2^. Therefore, these seven large size ratio triple asteroids are all hierarchically stable when without considering the solar gravity effect and primary’s *J*_2_.

**Table 1:** Ratios of masses and orbital radii for the seven known large size ratio triple asteroids

System	*μ*_2_	*μ*_3_	*μ*_4_	*α*_23_	*α*_34_	Primary’s *J*_2_
(45) Eugenia ^a,b^	4.45×10^−5^	4.45×10^−5^	3.53×10^11^	5.25×10^−1^	2.86×10^−6^	0.0581
(87) Sylvia ^c^	4.94×10^−5^	6.28×10^−5^	1.34×10^11^	5.21×10^−1^	2.60×10^−6^	0.09959
(93) Minerva ^d^	8.60×10^−6^	1.23×10^−5^	5.94×10^11^	6.01×10^−1^	1.51×10^−6^	0.08
(216) Kleopatra ^e^	1.40×10^−4^	3.00×10^−4^	4.29×10^11^	6.70×10^−1^	1.62×10^−6^	0.6
(136108) Haumea ^f^	4.47×10^−4^	4.50×10^−3^	4.97×10^8^	5.14×10^−1^	7.74×10^−6^	0.244
(136617) 1994 CC ^g^	2.24×10^−2^	3.50×10^−3^	7.67×10^18^	2.82×10^−1^	2.50×10^−8^	0.014
(153591) 2001 SN263^g^	1.07×10^−2^	2.62×10^−2^	2.17×10^17^	2.29×10^−1^	5.60×10^−8^	0.013

Note: The masses of (93) Minerva’s moonlets are estimated by assuming their spherical shapes and same density with the primary

^a^ Marchis et al. (2010). ^b^ Beauvalet et al. (2011). ^c^ Fang et al. (2012). ^d^ Marchis et al. (2013). ^e^ Descamps et al. (2011). ^f^ Ragozzine and Brown (2009). ^g^ Fang et al. (2011)

### With consideration of the solar gravity effect and primary’s *J*_2_

**Fig. 2:**
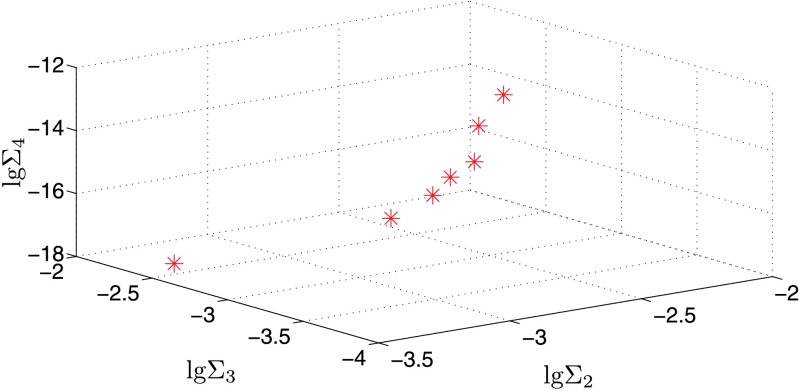
The values of empirical stability parameters *Σ*_2_, *Σ*_3_ and *Σ*_4_ for the seven known large size ratio triple asteroids when considering the solar gravity effect and primary’s *J*_2_. The actual large size ratio triple asteroids cases are marked with asterisk

When the solar gravity effect and primary’s *J*_2_ is taken into consideration, the case is a four-body problem, which is a bit more complicated. According to Eqs. (5)–(7), the values of *Σ*_2_, *Σ*_3_ and *Σ*_4_ for the seven known large size ratio triple asteroids are shown in Fig. 2. It can be seen that the values of *Σ*_2_, *Σ*_3_ and *Σ*_4_ are all much less than 10^−2^ for all the known high size ratio asteroids. It means that all the known high size ratio asteroids are hierarchically stable. This result is consistent with numerical investigations of (87) Sylvia (Winter et al. 2009; Frouard and Compère 2012; Fang et al. 2012), which showed that the triple system (87) Sylvia is orbitally stable.

## Conclusions

In the present paper, the hierarchical stability of the seven known large size ratio triple asteroids is analyzed. The solar gravity and primary’s *J*_2_ are considered. The force function for the four-body problem is expanded in terms of mass ratios based on the Hill’s approximation and the large size ratio property. The hierarchical stability of the triple asteroids is examined using the empirical stability parameters. Without considering the solar gravity and primary’s *J*_2_, it is found that all the seven known high size ratio asteroids are hierarchically stable. While considering the solar gravity and primary’s *J*_2_, all the known high size ratio asteroids are still hierarchically stable. We concluded that the hierarchical arrangement of the system will not be altered for (45) Eugenia, (87) Sylvia, (93) Minerva, (216) Kleopatra, (136617) 1994 CC, (153591) 2001 SN263, and (136108) Haumea over a long time.
